# A Protocol for Simultaneous In Vivo Imaging of Cardiac and Neuroinflammation in Dystrophin-Deficient MDX Mice Using [^18^F]FEPPA PET

**DOI:** 10.3390/ijms24087522

**Published:** 2023-04-19

**Authors:** Joanne M. Tang, Andrew McClennan, Linshan Liu, Jennifer Hadway, John A. Ronald, Justin W. Hicks, Lisa Hoffman, Udunna C. Anazodo

**Affiliations:** 1 Department of Medical Biophysics, Western University, London, ON N6A 3K7, Canada; 2 Lawson Health Research Institute, London, ON N6A 4V2, Canada; 3 Robarts Research Institute, Western University, London, ON N6A 3K7, Canada; 4 Department of Anatomy and Cell Biology, Western University, London, ON N6A 3K7, Canada; 5 Department of Neurology and Neurosurgery, Montreal Neurological Institute, McGill University, Montreal, QC H3A 0G4, Canada

**Keywords:** Duchenne muscular dystrophy, [^18^F]FEPPA, positron emission tomography (PET), cardiac inflammation, neuroinflammation, *mdx:utrn*(+/−) *mice*

## Abstract

Duchenne muscular dystrophy (DMD) is a neuromuscular disorder caused by dystrophin loss—notably within muscles and the central neurons system. DMD presents as cognitive weakness, progressive skeletal and cardiac muscle degeneration until pre-mature death from cardiac or respiratory failure. Innovative therapies have improved life expectancy; however, this is accompanied by increased late-onset heart failure and emergent cognitive degeneration. Thus, better assessment of dystrophic heart and brain pathophysiology is needed. Chronic inflammation is strongly associated with skeletal and cardiac muscle degeneration; however, neuroinflammation’s role is largely unknown in DMD despite being prevalent in other neurodegenerative diseases. Here, we present an inflammatory marker translocator protein (TSPO) positron emission tomography (PET) protocol for in vivo concomitant assessment of immune cell response in hearts and brains of a dystrophin-deficient mouse model [*mdx:utrn*(+/−)]. Preliminary analysis of whole-body PET imaging using the TSPO radiotracer, [^18^F]FEPPA in four *mdx:utrn*(+/−) and six wildtype mice are presented with ex vivo TSPO-immunofluorescence tissue staining. The *mdx:utrn*(+/−) mice showed significant elevations in heart and brain [^18^F]FEPPA activity, which correlated with increased ex vivo fluorescence intensity, highlighting the potential of TSPO-PET to simultaneously assess presence of cardiac and neuroinflammation in dystrophic heart and brain, as well as in several organs within a DMD model.

## 1. Introduction

Duchenne muscular dystrophy (DMD) is a progressive neuromuscular degenerative disease, affecting approximately 1 in 3600 live male births worldwide [1]. Individuals with DMD are unable to produce functional Dystrophin protein which is found systemically across various tissues, notably in skeletal and cardiac muscle, and neurons in the central nervous system (CNS). As a result, DMD is clinically characterized by progressive skeletal and cardiac muscle degeneration along with cognitive impairment [2,3,4]. These multi-organ degenerations are exacerbated by fibrosis, ischemia, and chronic inflammation until an early death from cardiac or respiratory complications [5,6,7]. Although there is still no cure for DMD, recent advancements in experimental therapies have prolonged both ambulation and life expectancy to 30–40 years [8,9].

With increased longevity in DMD patients, the clinical relevance of heart disease and cognitive impairment is becoming more apparent. More than 90% of DMD patients over the age of 18 show signs of cardiac involvement, with nearly 60% of DMD patients dying from cardiac complications by age 19 [9,10]. Currently, it is known that the dystrophin loss in cardiac muscles leads to membrane integrity destabilization of striated cardiac muscle fibers, which in turn contributes to increased intracellular calcium levels and subsequent muscle fiber deterioration [11,12]. As with skeletal muscle, it is suspected that this initiates the pathological cycle of chronic inflammation, fibrosis, and necrosis, leading to damage in regions of high contractility and movement (e.g., left ventricle). The loss of viable myocardium leads to further fibrosis, and the clinical emergence of cardiomyopathy and eventually heart failure [6,7,13,14,15].

In the brain, dystrophin plays a role in brain development and aging. The lack of intrinsic dystrophin gene products within CNS—namely Dp427, Dp140, and Dp71—are thought to contribute to cognitive weakness by causing functional and morphological abnormalities to occur [3,4,16]. However, the underlying mechanisms are not well-understood. Cognitive and behavioural symptoms usually manifest in the form of lowered intelligence quotient (IQ) scores, learning difficulties, memory deficits, and higher incidences of neuropsychiatric disorders [16]. Recent investigations also report the delayed emergence of cerebral infarcts and progressive cognitive decline within older DMD subjects, leading to the possible paradigm of neurodegeneration in the later stages of disease progression [13,17,18].

There is growing evidence that inflammation may be an inciting factor in skeletal and cardiac muscle degeneration in DMD. Inflammation is thought to exacerbate symptoms and promote muscular degeneration [5,7,14] and we have demonstrated that the degree of inflammatory cell infiltration is associated with disease progression within dystrophin-deficient murine models [19]. Despite neuroinflammation being a prime component of several pediatric and adult neurodegenerative disorders [20], the role of inflammation within the dystrophic brain is relatively unexplored. To the best of our knowledge, immune cell infiltration in the brain has yet to be demonstrated within DMD patients or dystrophin-deficient animals. However, in brain tissue from *mdx* mice, heightened levels of pro-inflammatory interleukin (IL)-1β and tumor necrosis factor (TNF)-α associated with several neurological diseases [21] have been found and several cognitive deficits have also been observed [22]. Considering the well-known consequences of unchecked inflammation potentially leading to cardiac and neurodegeneration, there is an unmet need to better understand the role of inflammation in multi-organ degeneration, as it may lead to the development of effective therapeutic strategies that targets multiple tissues systems especially brain and heart resilience in DMD patients.

**Figure 1:**
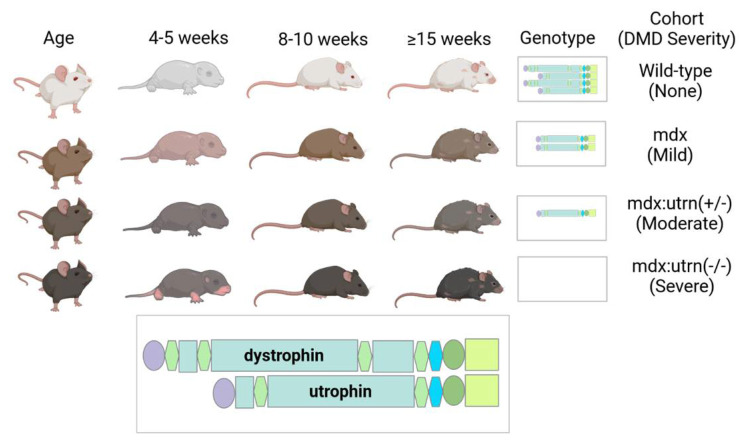
**Murine models of DMD** including the dystrophin-deficient mdx:utrn(+/−) mouse model used in this pilot study and the feasibility of modeling DMD symptom severity longitudinally with consideration for age-related effects.

Recent advancements in non-invasive molecular imaging techniques for assessing inflammatory load have inspired interest in understanding the role of inflammation in multi-organ degeneration in several disease systems. In particular, Thackeray et al. [23] demonstrated evidence of concomitant inflammation in both the hearts and brains of ischemic heart disease mice and patients using positron emission tomography (PET) imaging targeting mitochondrial translocator protein (TSPO). TSPOs are highly expressed on activated microglia and macrophages [24], making it a promising tool for in vivo multi-organ imaging when combined with positron emission tomography (PET). Thus, this exploratory study sought to probe the capacity of TSPO-PET imaging in assessing in vivo inflammatory involvement in the dystrophic heart and brain, and across several organs. Specifically, we used [^18^F]-N-(2-(2-fluoroethoxy)benzyl)-N-(4-phenoxypyridin-3-yl)acetamide ([^18^F]FEPPA), a second-generation TSPO tracer, to simultaneously assess cardiac and neural inflammation in a dystrophin-deficient *mdx:utrn*(+/−) mouse model with one functional utrophin protein (Figure 1), known to exhibit moderate to severe disease phenotypes that better mimic human cardiomyopathy symptoms [24]. Our primary aim was to design an experimental protocol to test the hypothesis that *mdx:utrn*(+/−) mice will have increased inflammation levels in dystrophic cardiac and neural tissues, which will be exhibited as heightened TSPO-PET signal and correlative histological TSPO expression.

## 2. Results

**Figure 2:**
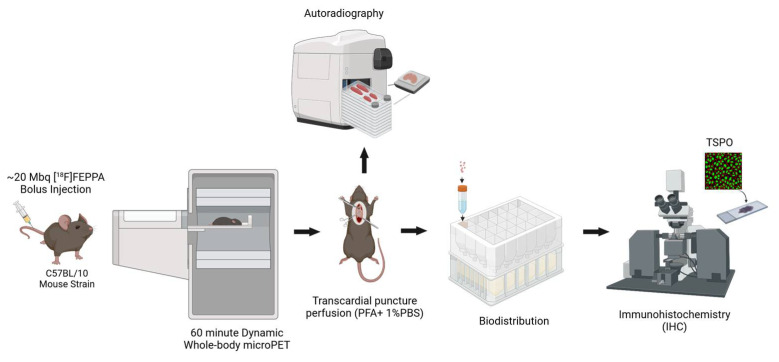
**Experimental protocol for simultaneous in vivo [^18^F]FEPPA PET imaging and ex vivo histopathology confirmation** for assessing multi-organ inflammatory involvement in DMD mice models. PFA = paraformaldehyde; PBS = phosphate-buffered saline; TSPO = (18 kDa) translocator protein.

**Figure 3:**
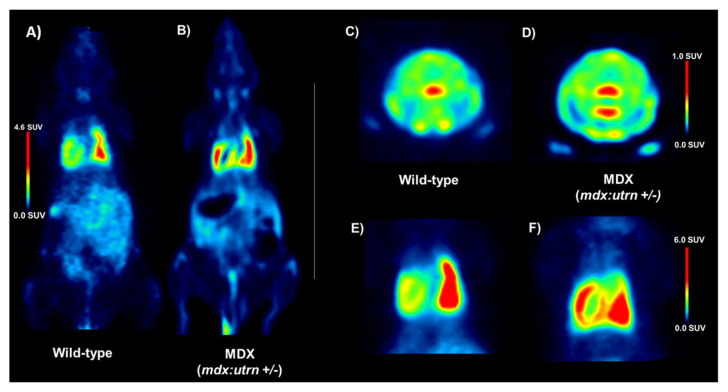
**[^18^F]FEPPA SUV images of representative 8–10 week wild-type (A,C,E) and age-matched MDX dystrophy (B,D,F) mice**. Coronal whole-body (**A**,**B**), axial whole brain slices (**C**,**D**), and heart images (**E**,**F**) were generated from PET time-activity curves at 30–60 min (*n* = 4–6 mice/genotype). MDX = dystrophin-deficient *mdx:utrn*(+/−) mouse model, SUV = standardized uptake values.

**Figure A1:**
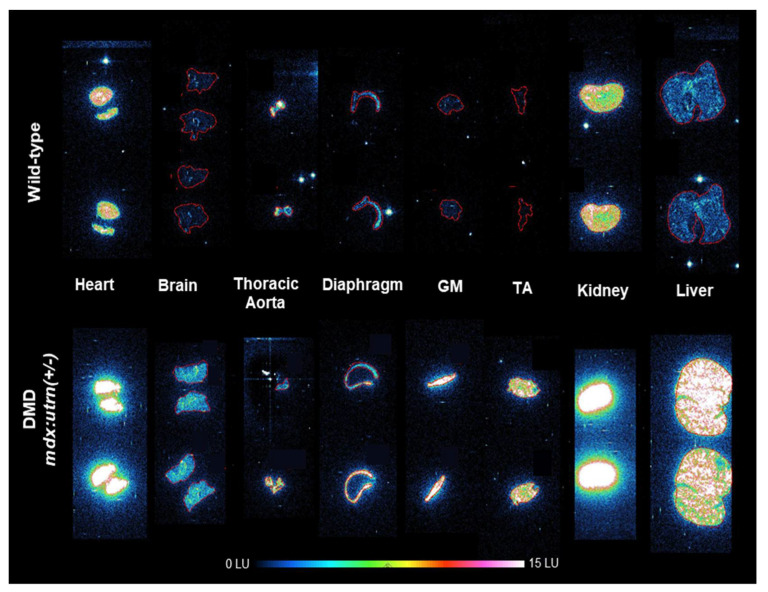
Autoradiography images of the heart, brain, and multiple tissues excised from a representative MDX and wild-type mice injected with [^18^F]FEPPA. LU = light units. MDX = dystrophin-deficient *mdx:utrn*(+/−) mouse models.

**Figure A2:**
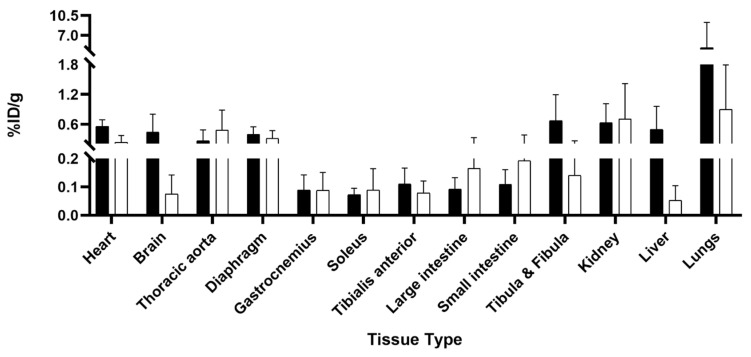
Multi-organ Biodistribution of [^18^F]FEPPA within murine models. Data (±SE) shows the differences in radiotracer uptake as across multiple tissues as the percentage of the injected dose per gram of the tissue (%ID/g) of MDX (*mdx:utrn*(+/−)) (black bars; *n* = 4 mice) and age-matched wild-type controls (white bars; *n* = 2 mice). No statistical analyses were conducted due to low sample sizes.

**Figure A3:**
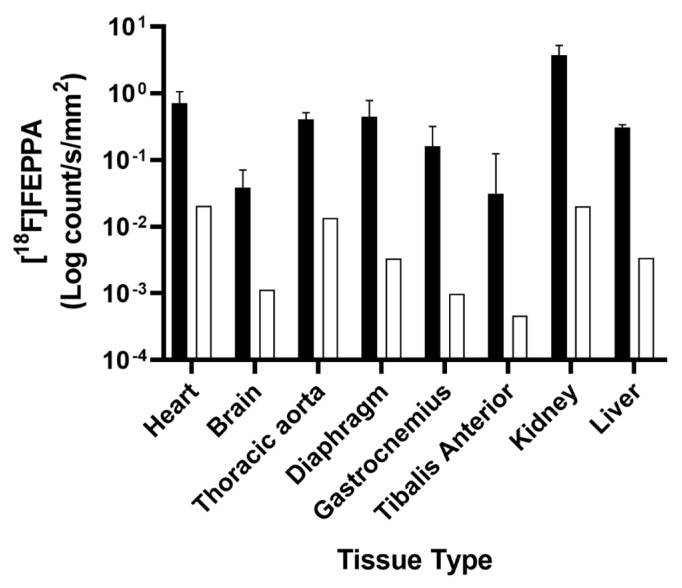
Quantified autoradiography of [^18^F]FEPPA activity in MDX and wild-type mice. Data (±SE) depicts the log counts per second per millimetre squared area of [^18^F]FEPPA activity within slices of heart, brain, thoracic aorta, diaphragm, skeletal muscles, kidney and liver for MDX (black bars; *n* = 3 mice) and an age-matched wild-type control (white bars; *n* = 1 mouse). No statistical analyses were conducted due to low sample sizes. MDX = dystrophin-deficient *mdx:utrn*(+/−) mouse models.

We explored our experimental design and imaging approach (Figure 2) in a pilot study of four *mdx:utrn*(+/−) (MDX) (two females, two males) and six wild-type (WT) (two females, four males) mice aged 8–10 weeks old to estimate the sample size to adequately test the hypothesis. All mice were both able to take up [^18^F]FEPPA throughout the entire body—notably binding to our tissues of interest, the heart and brain (Figure 3). Although there were not sufficient tissue samples to measure differences in [^18^F]FEPPA binding using autoradiography and biodistribution, the preliminary results demonstrate [^18^F]FEPPA activity occurring body-wide within the heart and brain as well as in several other tissues, such as skeletal muscles, aorta, diaphragm, etc., as shown in Appendix A Figure A1, Figure A2 and Figure A3.

### 2.1. Elevation of In Vivo Inflammation-Targeted Radiotracer Binding in DMD Models

**Figure 4:**
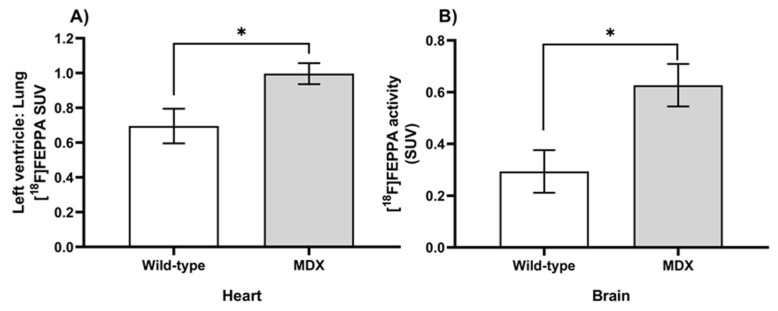
**Quantified [^18^F]FEPPA activity in hearts (A) and brains (B) of age-matched MDX and wild-type mice.** Standardized uptake values were generated from PET time-activity curves at 30–60 min using manually drawn regions of interest (ROIs) segmented for the left ventricle, lung, and whole brain (*n* = 3–4 slices/mouse/genotype). Significant differences (*p* < 0.05; indicated by *) were observed between wild-type (white bars) and *mdx:utrn*(+/−) mice (gray bars) for both left ventricle-to-lung ratio (**A**) and whole brain (**B**) SUVs using Welch’s two-way *t*-test. Data are depicted as mean ± standard error. TSPO = (18 kDa) Translocator protein, MDX = dystrophin-deficient *mdx:utrn*(+/−) mouse model, SUV = standardized uptake values.

To assess the influence of inflammation on dystrophic cardiac and neural tissue, inflammation was quantified from the [^18^F]FEPPA PET images as mean standard uptake values (SUV). In the thoracic region, left-ventricle-to-lung mean SUV ratios indicated that MDX mice had significantly higher [^18^F]FEPPA uptake (Figure 4; t = 2.58, *p* = 0.0338), as these left-ventricle-to-lung ratios increased from 0.63 ± 0.10 in WT mice to 0.99 ± 0.06 in the MDX cohort. In neural tissue, similar accumulations of inflammatory tracer were also observed in MDX mice. The WT brains demonstrated [^18^F]FEPPA uptake of 0.34 ± 0.08 SUV, while MDX brains experienced 82.4% more uptake at 0.62 ± 0.08 SUV.

### 2.2. Ex Vivo TSPO Signal Indicates Heightened Cardiac and Neuroinflammation in DMD

**Figure 5:**
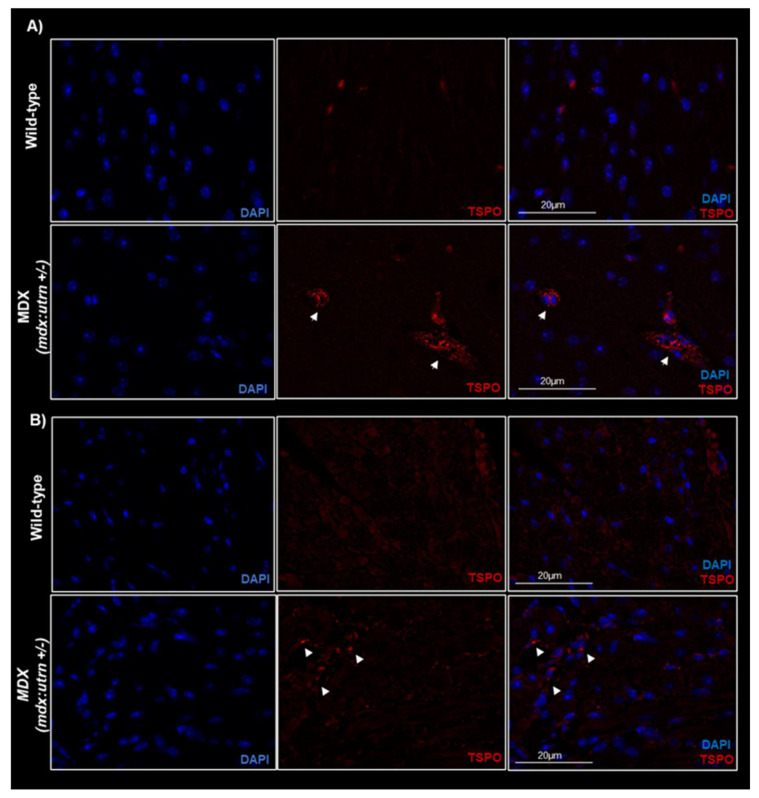
**Ex vivo histology of TSPO-bound microglial and macrophages in an MDX and wild-type mice cardiac (A) and neural tissues (B).** Representative fluorescence immunostained images of microglial and macrophages with TSPO (red) and DAPI (blue) in 8–10 weeks old *mdx:utrn*(+/−) mice depict qualitatively more prevalent TSPO expression in the dystrophin-deficient mice. White arrow heads indicate regions of TSPO signal. Scale bar overlaid on merged (DAPI and TSPO) images = 20 μm in length. Translocator protein, MDX = dystrophin-deficient *mdx:utrn*(+/−) mouse model.

**Figure 6:**
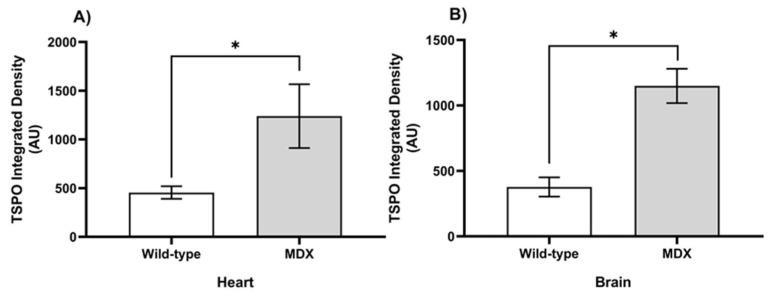
**Quantified fluorescence immunohistochemical images of microglial and macrophages with TSPO in 8–10 weeks old MDX and wild-type subjects**. Data (mean ± SE) depict higher TSPO in *mdx:utrn*(+/−) mice (gray bars) than wild-type controls (white bars) (*n* = 3 mice/genotype). Significant differences (*p* < 0.05), indicated by *, were observed for slices of cardiac (**A**) and neural (**B**) tissue when compared using Welch’s two-way *t*-test (*n* = 5–10 images/mouse/genotype). AU = arbitrary units. Other abbreviations as described in prior figures.

**Figure 7:**
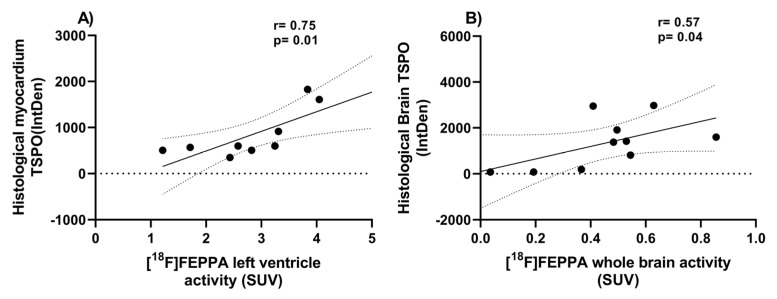
**Correlation of [^18^F]FEPPA activity with histological TSPO fluorescence intensity.** Data depict the strong correlation of the left ventricle (**A**) or whole brain (**B**) PET avtivity, with quantified TSPO myocardium or neural tissue fluorescence signal. Pearson product-moment correlation coefficients (r) were calculated on all animals imaged with PET (*n* = 9 for heart association with 1 outlier excluded and *n* = 10 for brain associations, no outlier detected). Significance was considered when *p* < 0.05. IntDen = Integrated Density, the product of the mean fluorescence intensity and area of selected cell.

**Figure A4:**
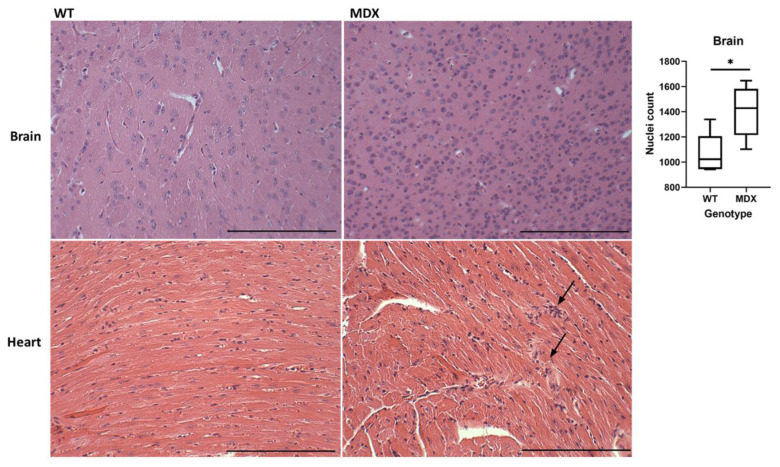
Representative Hematoxylin and Eosin (H&E) stained images from heart and brain tissue sections of an 8–10 week dystrophin-deficient *mdx:utrn*(+/−) mouse (MDX) and age-matched C57bl/10 wild-type (WT) mouse. Heart tissue obtained from MDX mouse show infiltrate (black arrows) within heart tissue and higher nuclei counts (mean (±SE)) in brain tissues. Heart image filter chosen to highlight myocardial fibers. Images scale bar = 100 µm; one biological and 3 technical replicates were used for the heart and one biological, and 6 technical replicates were used for the brain. Significant difference (*p* < 0.05), indicated by *.

Fluorescence immunostaining of heart and brain slices demonstrated the presence of TSPO in age-matched MDX mice. Although both groups expressed a modest baseline level of TSPO (Figure 5), MDX mice consistently expressed significantly higher TSPO fluorescence intensity in both cardiac (Figure 6; t = 2.35, *p* = 0.025) and neural tissue (Figure 6; t = 5.15, *p* < 0.001). Within cardiac tissue, MDX mice experienced a 63.9% increase in fluorescence intensity when compared to age-matched wild-type mice; as dystrophic hearts demonstrated TSPO fluorescence intensities of 1261.57 ± 307.76 arbitrary unit (AU) compared to those of wild-type mice at 454.59 ± 63.93 AU. Similarly, in neural tissue, TSPO fluorescence intensity was remarkably 68.3% lower in WT compared to MDX mice. Dystrophic brain tissues displayed fluorescence intensities of 1149.74 ± 148.35 AU, which is lower than the 364.84 ± 73.71 AU observed in WT brains. Histological myocardium TSPO signal significantly correlated with [^18^F]FEPPA uptake in the left ventricle (Figure 7; r = 0.75, *p* = 0.01). This was also observed between neural tissue TSPO fluorescence intensity in histology and in vivo whole brain TSPO-PET tracer SUV values (Figure 7; r = 0.57, *p* = 0.042). Analysis of the H&E staining (Appendix A, Figure A4) showed infiltration within heart tissue and higher nuclei counts in the MDX brain compared to age-matched WT mice (t = 3.17, *p* = 0.01).

### 2.3. Sample Size Estimation

Given that findings from cardiac tissue fluorescence immunostaining showed the least between group differences in TSPO levels across modalities and in comparison, to the brain, and more importantly required ex vivo analysis, its results were used to estimate the number of samples per group required to detect the minimum difference in TSPO signal for a larger scale study. A minimum of seven 8–10 weeks old *mdx:utrn*(+/−) and seven age-matched WT mice are required to detect at least 63% differences in TSPO levels.

## 3. Discussion

The goal of this exploratory study was to evaluate [^18^F]FEPPA PET as a tool to assess inflammation in vivo in multiple organs of mice with dystrophic disease. We found 8–10 weeks old dystrophin-deficient mice had elevated [^18^F]FEPPA uptake in cardiac and neural tissues compared to healthy controls, which mirrored heightened ex vivo TSPO levels in our histological data. These results, while preliminary support our hypothesis that subjects with dystrophic deficiency demonstrate significant inflammation in their heart and brains, which can be confirmed in a larger experimental study using TSPO-PET.

To the best of our knowledge, our study is the first to observe significantly elevated TSPO-PET in the heart and brains of MDX mice. Interestingly, these increases seem to be occurring body-wide within several other tissues, akin to other diseases linked to chronic inflammation (e.g., atherosclerosis, myocardial infarctions, etc.) [25,26,27]. As such, it is likely that this heightened TSPO activity may be a consequence of activated macrophages and microglia within regions of tissue injury or dysfunction [27]. For example, immunohistostaining of mice one-week post-myocardial infarction indicated colocalization of TSPO to CD68+ microglia and cardiac monocytes within the brain cortex and infarcted myocardium respectively [25]. Interestingly, no colocalization was found between TSPO and GFAP-stained astrocytes at that time point. TSPO-tracers were also found to localize to magnetic resonance imaging (MRI)-identified ischemic lesions within ischemic stroke patients, further demonstrating the tracer’s feasibility to map inflammation after tissue damage [28]. Thus, as DMD pathophysiology is known to be associated with contraction-induced damage and severe immune cell infiltration, it is likely that TSPO is upregulated within regions of injury—manifesting as the displayed [^18^F]FEPPA tracer uptake within the dystrophic heart and brain. Our preliminary biodistribution and autoradiography observations support these claims, as the TSPO-tracer accumulated notably within regions associated with dystrophic symptoms (i.e., heart, brain, skeletal muscles, etc.). Although this exploratory study demonstrated the feasibility of [^18^F]FEPPA PET to assess in vivo inflammation simultaneously within the heart and the brain, our study cannot provide a definite answer to whether [^18^F]FEPPA PET can demonstrate inflammatory load within other tissues—despite the promising trends—due to the small sample sizes of our autoradiography and biodistribution data. To answer this question, a study using a larger sample size of seven or more mice per group along with quantification of tracer uptake in the other tissues within the PET data is needed.

In contrast to our results, three previous 2-deoxy-2-[^18^F]fluoro-D-glucose ([^18^F]FDG) PET studies reported lower mean cardiac SUV and highlighted select neural regions of hypometabolism in canine models of DMD [29,30,31]. While [^18^F]FDG can be used as an analogue of inflammation in several neurological and cardiac diseases, it should be noted that these specific studies were focused on investigating the metabolic functionality of dystrophin-deficient tissue regions rather than its associated peripheral inflammation [32,33]. Because of the heart and the brain’s disposition as highly metabolically-active organs, there is naturally a higher accumulation of [^18^F]FDG tracer within those regions, which makes it difficult to detect inflammatory infiltrates without being potentially obscured by background activity or alterations in myocardial/neuronal function [34,35]. Additionally, a multi-tracer study longitudinally tracking microglial activation and glucose hypometabolism simultaneously in a transgenic mouse model of Alzheimer’s disease observed discrepancies between the data trend of the TSPO-tracer [^18^F]GE-180 and [^18^F]FDG [36]. The authors observed progressive increases in [^18^F]GE-180 uptake throughout the entire course of the disease (5–16 months), which differed from the life-course kinetics of [^18^F]FDG which peaked at ~8 months of age before decreasing for the remaining 8 months, suggesting that the incidences of hypometabolism demonstrated within the dementia animals are occurring much later in life and as disease progressed. Interestingly, this early hypermetabolism may be capturing increased glial activity as it matches the peak of [^18^F]GE-180 at 8 months—indicating the potential early onset of inflammation prior to rampant hypometabolism (and with it, the neurodegenerative symptoms) [37]. Taken together, although prior [^18^F]FDG studies in DMD models show known indications of late-life brain and cardiac degeneration/dysfunction, our data highlight the potential role of inflammation in contributing to these metabolic deficiencies within dystrophin-deficient mice. Further longitudinal multi-tracer studies on dystrophic animal models using both [^18^F]FEPPA and [^18^F]FDG would greatly improve our understanding of the time course and interaction of inflammation and glucose hypometabolism onset. 

The increased in vivo [^18^F]FEPPA activity within the heart of our 8–10 weeks old MDX mice correlated with increases in ex vivo histology. We suspect that the [^18^F]FEPPA uptake is localized to the activated macrophages present in the dystrophic myocardium, increasing TSPO expression above baseline. Thus, making [^18^F]FEPPA a viable candidate for PET imaging of cardiac inflammation. Although we acknowledge that TSPO is constitutively expressed within cardiac tissue, the mRNA profile of TSPO typically remains at a steady moderate state within normal healthy tissue [38,39]. Importantly, TSPO is found to be overexpressed in inflammatory cardiac foci, seemingly being upregulated in activated immune cells [40,41]. TSPO-PET has similarly been used as a marker of cardiac macrophage infiltration in previous studies of myocarditis, and myocardial infarction [25,40]. As such, it is suggested that these heightened [^18^F]FEPPA activities may indicate activated macrophage presence within the murine dystrophic heart. 

Our data, highlighting the presence of TSPO-bound ligands in MDX mice and the histological evidence of M1-like (proinflammatory) and M2-like (reparative) macrophage infiltration into the sites of dystrophin-related injury support this hypothesis [42,43,44,45]. Interestingly, these suspected elevations in inflammatory load are observed quite early at 8–10 weeks—when the *mdx:utrn*(+/−) model heart function is relatively stable. This agrees with earlier reports that indicate a certain degree of inflammation, cellular necrosis, and fibrosis within their myocardium at 10 weeks of age [45]. However, it should be noted that one cardiac dystrophin-deficient murine study found a lack of macrophage infiltration into cardiac tissue until 6 months of age—in contrast to our results [46]. This delayed inflammatory onset might be due to the authors’ use of a comparatively less severe *mdx* model than ours, as it is known to demonstrate minimal—if any—cardiac dysfunction [47,48]. Within studies pertaining the same murine model—*mdx:utrn*(+/−)—evidence of ventricular dysfunction (i.e. impaired stroke volume, decreased ejection fraction, and elevated heart rate) were observed far later at 10 months, compared to our observed onset of cardiac inflammation at 8–10 weeks [49]. Thus, the present findings may indicate an early inflammatory onset prior to the onset of cardiac symptoms. Considering that *mdx:utrn*(+/−) mice who were started on an anti-inflammatory quercetin-enriched diet at 8 weeks old have comparatively minimal cardiac damage than those without, the early detection and intervention to modulate cardiac inflammation may be vital in possibly attenuating downstream DMD cardiac degenerative symptoms [50]. A more extensive explanation of this mechanism is outside of the scope of this paper. However, a further longitudinal study pairing this [^18^F]FEPPA PET protocol with an anatomical or morphological modality (such as MRI), may be undertaken to better assess how this early inflammatory response may contribute to dystrophic cardiac tissue pathology.

In vivo [^18^F]FEPPA SUV has been correlated with post-mortem pro-inflammatory markers histologically in other neuroinflammatory or disease models, validating its use as an analogue of activated microglia cells [51]. In this study, in vivo [^18^F]FEPPA signal within the whole brain correlated with ex vivo TSPO immunofluorescence intensity, suggesting the possible localization of activated microglia to the dystrophic neural tissue. To the best of our knowledge, there are no other studies demonstrating the presence of immune cell infiltration into the dystrophic brain. However, in support of our observations, heightened levels of IL-1β and TNF-α have been found within *mdx* murine brains, who also displayed cognitive impairment similar to those in DMD patients [22]. Recent literature has also speculated that these specific cytokines may participate in the emergence of DMD cognitive dysfunction symptoms by altering several features in synaptic transmission (see review in Rae and O’Malley [52] and Stephenson et al. [53]. Considering that activated microglia are known to upregulate TSPO expression and release pro-inflammatory cytokines (e.g., IL-1β, IL-6, TNF-α), it is possible that activated microglia may contribute to the dystrophic brain’s impairment [25,54,55]. Additionally, the activated microglia may also predict cognitive deterioration [56] as multiple studies regarding neurodegenerative diseases (e.g., Alzheimer’s dementia) reported that the degree of neuroinflammation can predict longitudinal cognitive decline [57]. While the underlying mechanism on how activated microglia contributes to downstream neurodegeneration is still under debate, the early observation of neuroinflammation within our study and the knowledge of late-onset cognitive decline within both older DMD patients (occurring at 30 years of age) [58] and aged murine models (occurring at 18 months) [17,22] suggests a possible similar association. As such, a future study using TSPO-PET to longitudinally assess neuroinflammation in these MDX murine models—alongside cognitive testing—is suggested to better delineate the relationship between early neuroinflammation and late-onset cognitive decline.

A possible explanation for this microglia activation within the brain may, in part, be due to pro-inflammatory cytokines—which are abundant in DMD circulation—passing through the “leaky” dystrophin-deficient blood-brain barrier (BBB) [59]. Within the brain, dystrophin—specifically Dp71—is located in the perivascular end-feet of astrocytes, normally participating in the stabilization and regulation of molecules transporting through the BBB [60]. In *mdx* mice, the reduction in Dp71 demonstrates severe alteration of endothelial and glial cells, as well as a reduction in the expression of zonula occludens and Aquaporin-4. As a result, *mdx* mice shows increased vascular permeability and by proxy, increased BBB permeability [60,61,62]. Increased BBB permeability and IL-6 levels within the brain were shown when systemic inflammation was induced by a peripheral lipopolysaccharide injection to an Alzheimer’s APP transgenic mouse, resulting in more severe cognitive symptoms [63]. Thus we speculate that the nature of this neural [^18^F]FEPPA uptake and subsequent TSPO overexpression may be a result of a similar incidence. However, it should be noted that within a study investigating the permeability of the BBB in *mdx* mice, CD4-, CD8-, CD20- and CD68-positive cells were not histologically observed within the BBB perivascular stroma [64]. These observations, while contrasting, do not conflict with our findings as alternate passages of cytokines through the BBB has already been extensively investigated [65,66]. A possible source of systemic inflammation/pro-inflammatory cytokines within our study include the dystrophin-deficient cardiac and skeletal muscles—which as stated before are known to sustain critical damage upon sarcolemma contraction and release pro-inflammatory cytokines into circulation. The concomitant cardiac and neural TSPO-tracer uptake and our autoradiography and biodistribution results (i.e. trends towards heightened binding across almost all tissue types) speaks to the systemic nature of inflammation within a dystrophin-deficient disorder, while hinting its possible contribution to both downstream cardiac and neurodegeneration damage. It will be interesting to investigate if the neuroinflammation observed in this murine model of DMD is widespread across the whole brain, or specific to certain regions, especially in the hippocampus where atrophy within this region has been linked to progressive cognitive impairment in *mdx* mice [17]. Further PET/MRI studies linking [^18^F]FEPPA PET to regional MRI volumetry and functional MRI network changes will help shed more light. 

The strengths of our proposed protocol include: (1) the utility of non-invasive inflammation imaging using second generation TSPO radioligand [^18^F]FEPPA, (2) the use of a high-resolution (1.4–1.5 mm) small animal PET scanner capable of multi-organ/whole-body image data acquisition, and (3) the showcase of tissue-specific dosimetry and molecular colocalization capabilities via biodistribution and autoradiography respectively in a murine model of DMD—allowing for the additional in vitro histopathological validation of our in vivo imaging studies. While the pilot study demonstrated that our imaging approach is well-tolerated and may not be burdensome for longitudinal studies across age groups, the pilot study does have several limitations. Firstly, the study sample size (Appendix B) is relatively small and uses a mixed sex murine cohort despite DMD being a X-linked genetic disorder and thus, primarily appearing in only human male patients. While this is a common notion in DMD pre-clinical literature since both sexes can express this phenotype through mutations in their dystrophic and utrophin genes, there have been reports that female patients and rodents express constitutively higher levels of TSPO in both cardiac and neural tissue [48,67,68,69]. Due to low sample sizes, we could not account for these potential sex differences with this study; however, a fair balance between sexes were used (wild-type: 2 females, 4 males; MDX: 2 females, 2 males). This relatively small cohort could underpower our sample size estimation since analysis of covariates such as sex differences were not included in the sample size analysis. The use of male-only or female-only mice for the larger study could reduce the likelihood of underpowered studies. Consequently, future studies using same-sex subjects are strongly recommended. Secondly, similar to other TSPO-PET literature, [^18^F]FEPPA is unable to differentiate between macrophage/microglia morphological states (i.e. pro-inflammatory and anti-inflammatory). Further studies including in-depth histopathological analysis using H&E staining and immunohistochemistry of dedicated inflammatory antibodies (CD68 or F4-80) co-localized to TSPO could reveal contributions of pro-inflammatory macrophage and microglia phenotypes, as well as the extent of inflammation in MDX heart and brain tissues. Lastly, while [^18^F]FEPPA is commonly used to assess activated microglia, it is difficult to discern the true sensitivity of this tracer within DMD as TSPO is also present in astrocytes, pericytes, and endothelial cells, among other structures within the brain at low levels and within injured cardiomyocytes. During DMD, astrocytes may be activated due to a lack of functional dystrophin, as its absence can precipitate a series of complex signaling cascades that leads to glutamate toxicity in the CNS [70]. However, it should be noted that the percentage of cells expressing TSPO are reported to be ~7 times higher for microglia than for astrocytes, as measured by scRNA-seq—suggesting a preference for the TSPO radiotracer to bind to microglia [71]. Thus, it is suggested that further multi-tracer studies using both [^18^F]FEPPA and specific PET tracers targeting solely activated macrophages or microglia such as triggering receptor expressed on myeloid cells (TREM) can be used to further validate the use of [^18^F]FEPPA as a multi-organ inflammation assessment tool in DMD [72].

## 4. Materials and Methods

The pilot study for protocol development of a larger scale study was conducted at Lawson Health Research Institute at St. Joseph’s Health Care in London, Ontario, Canada. All animal protocols were approved by the Animal Use Subcommittee at Western University, London, Ontario, Canada and were conducted in accordance with guidelines set by the Canadian Council on Animal Care (CCAC).

### 4.1. Study Population

Breeding pairs of wild-type and functional dystrophin-deficient mdx:utrn(+/−) (a point mutation in dystrophin gene and a single utrophin allele lost) mice [73] were purchased from Charles River and Jackson Laboratories (Bar Harbor, ME, USA). The C57BL/10 (Jax stock #000665) substrain widely used in immunological research were used as wild-type while the mdx:utrn(+/−) mice were on a C57BL/10ScSnJ genetic background (Jax stock #000476). The C57BL/10ScSnJ substrain are similar to C57BL/10 except for minor known behavioural differences and a propensity for lower brain glutamic acid decarboxylase [74,75]. However, there is no evidence that the C57BL/10ScSnJ strain have altered inflammatory response and unlike the C57BL/c10, both strains are not known to have the spontaneous Toll-like receptor 4 (Tlr4) deletion that could produce hyposensitivity to microglia/macrophage [76]. Colonies were maintained under controlled conditions (19–23 °C, 12-h light/dark cycles), and were allowed water and food ad libitum. Two separate groups of eight- to ten-week-old mice were used in this study where the in vivo imaging (*n* = 4–6 mice/genotype) and ex vivo histology cohorts (*n* = 3 mice/genotype) are the PET and immunohistochemistry (IHC) cohort, respectively.

### 4.2. PET Imaging Protocol

All PET mice were induced in a chamber with 3% oxygen-balanced isoflurane mixture and then anesthetized with 1.5–2%; both mixtures were delivered at a constant rate of 1 L/min via a nose cone. After induction, these mice were imaged using a micro-PET scanner (eXplore VISTA, GE Healthcare, Chicago, IL, USA; Inveon DPET, Siemens, Munich, Germany). To assess whole-body inflammation accumulation, TSPO-targeted PET images were obtained after [^18^F]FEPPA tracer injection. 30 s following the start of the scan, a dose of approximately 20 MBq of prepared [^18^F]FEPPA in saline (approximately 5 μg/kg) was administered via tail vein catheter for dynamic acquisition. Summarily, 60-min whole-body dynamic scans in list-mode were acquired using the Inveon system or the eXplore VISTA scanner. Because of the limited field-of-view of the eXplore VISTA scanner not fully encompassing the whole mice, dynamic imaging was performed from head-to-chest followed by a 30-min full-body static scan. Injected dose did not exceed 0.3 mL to ensure proper animal health conditions. 

### 4.3. PET Image Analysis

For each mouse, the dynamic PET list mode data were reconstructed into the following time frames: 12 × 10 s, 60 × 30 s, 5 × 60 s, 5 × 120 s, 8 × 300 s using ordered subset expectation maximization (OSEM, Shoham, Israel) algorithm with no scatter and attenuation correction. Data were corrected to injected dose and decay corrected to start of PET scan using in-house MATLAB v2019a scripts (Mathworks, Natick, MA, USA). Standardized Uptake Values (SUV) were generated from PET data 30–60 min post-injection in PMOD 3.9 (PMOD Technologies, Zurich, Switzerland). Using manually drawn regions of interest (ROI), mean SUV were calculated for the left ventricle, lung, and whole brain covering 3–4 slices spanning each whole organ. Left ventricle-to-heart ratio—used to offset lung [^18^F]FEPPA activity and act as a correlative of cardiac events—was calculated from mean SUV within each animal [77].

### 4.4. Biodistribution and Autoradiography

The mice were sacrificed immediately after imaging through 5% oxygen-balanced isoflurane gas euthanasia followed by cervical dislocation. To preserve tissue anatomy, the mice underwent whole animal perfusion fixation via an intracardiac infusion of 4% paraformaldehyde (Sigma-Aldrich), and then phosphate-buffered saline (PBS) as directed in Gage et al. [78] before the heart and brain were dissected. Each heart was bisected twice—once transversely and once along the septum—and each brain were bisected along the central sulcus to ensure that exactly half of each tissue was fixed in 10% Formalin or frozen in Optimal Cutting Temperature solution (VWR) for biodistribution and autoradiography use respectively. Biodistribution was conducted for the heart, brain, thoracic aorta, diaphragm, gastrocnemius, soleus, tibialis anterior, large/small intestines, tibia/fibula, kidney, liver, and lungs; each organ was weighed for quantitative estimation of gamma counts from the ^18^F conjugate using the ORETC DSPEC50 Spectrometer. Radioactivity obtained from different organs was calculated as the percentage of the injected dose per gram of the tissue (%ID/g) and decay corrected to time of injection. Radioactivity was standardized to the dose injected into each animal. For autoradiography, frozen tissue samples were cryosectioned into a thickness of 20 μm with a Leica Clinical Cryostat (CM1850, Leica Biosystems, Wetzlar, Germany). Autoradiographic images of the heart, brain, thoracic aorta, diaphragm, gastrocnemius, tibialis anterior, kidney and liver were acquired for 12 h using a digital autoradiography system (BeaQuant AI4R, Nantes, France) fitted with a positron holder.

### 4.5. Histology Tissue Preparation

To supplement data from acute imaging for immunohistochemistry and histopathological analysis, a cohort of 8–10-week-old mice were sacrificed through cervical dislocation following CO_2_ gas euthanasia without PET imaging. The heart and brain were dissected and fixed in 10% formalin for 24–48 h. These tissues were embedded in paraffin for immunohistochemistry and histopathology by the Molecular Pathology facility (Robarts Research Institute, London, ON, Canada) and cut into 10 μm thick sections. Care was taken to ensure that the tissues were embedded in the same orientation within each block.

### 4.6. Immunohistochemistry Protocol

Following a modified protocol based on Abcam standards, tissue sections were deparaffinized and rehydrated in a series of xylene and ethanol washes prior to heat-mediated antigen retrieval in a citrate buffer for 30 min. Slides were then cooled slowly to room temperature, and Background Sniper (Biocare Medical, Concord, CA, USA) was applied for 8 min to reduce nonspecific background staining. Sections were incubated overnight at 4 °C with either primary anti-PBR (1:200, Abcam), primary anti-α-SMA (1:500, Abcam), or no antibodies—the latter acting as the positive and negative control. All antibodies were diluted in 1% bovine serum albumin (BSA) PBS. Following thorough washing with 1 × PBS, Alexafluor IgG (Life Technologies, 1:500) secondary antibodies were used to visualize the primary antibodies: anti-PBR sections were incubated with 594 Goat anti-rabbit IgG, and anti-α-SMA sections with 488 Goat anti-mouse IgG for 2 h at room temperature. For heart tissue, a solution of Cu_2_SO_4_•5H_2_O (10 mM copper sulfate, 50 mM ammonium acetate buffer, pH 5.0) was applied thereafter to prevent red blood cell autofluorescence. Additional 1 × PBS washes and an immersion in 0.1% Sudan Black B was performed to quench autofluorescence in both heart and brain tissue sections. Lastly, ProLong Gold anti-fade with DAPI (Life Technologies) was added to all sections to visualize the nuclei and to mount the coverslips onto glass slides. 

### 4.7. Microscopy and Image Analysis

Fluorescent images were acquired on an epifluorescence microscope (Nikon Eclipse Ts2R) using NIS Elements Microscope Image Software. Non-overlapping fields of view at 60x magnification were taken for each tissue section (*n* = 5–10 images/slide). Quantitative assessment of TSPO fluorescent signal in both wild-type and *mdx:utrn*(+/−) (henceforth named MDX) mice —while minimizing image exposure and auto-fluorescence (i.e. background signal)—was performed using an in-house semi-automatic grey scale thresholding protocol in ImageJ (LOCI, Madison, WI, USA) with FIJI package v2.0.0 [29].

### 4.8. Hematoxylin and Eosin (H&E) Staining

Routine H&E staining were applied on deparaffinized heart and brain tissue samples cut to 5 µm slices to show the extent of inflammation and visualize changes in tissue morphology. H&E stained images were captured using a Zeiss Axioskop Fluorescence microscope (Carl Zeiss Jena GmbH, Jena, Germany) and nuclei counts were determined using ImageJ [79].

### 4.9. Statistical Analysis

Analyses were performed using RStudio v1.0.136 (Boston, MA, USA) or SPSS 26 (IBM, Armonk, NY, USA) software. Data results are expressed as mean ± standard error (SE). Comparisons between groups were performed using Welch’s two-tailed *t*-test. No statistical analyses were conducted on autoradiography and biodistribution data due to low sample sizes. Negative biodistribution values for which measurement errors resulted in negative tissue weights (i.e., stemming from weight of empty tube exceeding the weight of tube and tissue) were removed from the data set. One-tailed Pearson correlation coefficients were calculated between [^18^F]FEPPA left ventricle/whole brain uptake, and myocardium/neural tissue histological TSPO fluorescence intensity using GraphPad Prism version 9.3.1 for Windows (San Diego, CA, USA). Outliers were identified using the ROUT coefficient Q method implemented in GraphPad Prism and excluded from the correlational analysis. *p*-values of less than 0.05 were considered significant. Replicate numbers are indicated in the figure legends.

### 4.10. Sample Size Estimation

The sample size for a larger scale study was estimated using the *p*-value method [80] for two independent samples, as described in the equation below.

$$N={\left[\left(\frac{Z_{\pi }\pm Z_{1-\frac{\alpha }{2}}}{Z_{1-\frac{P}{2}}}\right)\right]}^{2}\;N_{ref}$$

where *Z*_π_ is the z-value from the standard normal distribution for power (π) of 80% = 0.84, and *Z*_1−α/2_ is the z-value from the standard normal distribution for the two-sided significance level (α) of 0.05 = 1.96, *Z*_1−*P*/2_ is the z-value from the standard normal distribution for the *p*-value from the between group comparison performed using the Welsh’s two-tailed *t*-test on the pilot study data. The between group comparison of the fluorescence immunostaining of the heart slices was used, since it showed the least effect (minimum detectable difference) compared to PET or autoradiography findings. Based on this, *Z*_1−*P*/2_ for the *p*-value of 0.025 = 2.24.

## 5. Conclusions

In general, this exploratory study suggested that dystrophin-deficient mice were associated with higher inflammatory [^18^F]FEPPA radiotracer binding, mirroring ex vivo histological TSPO data within both their hearts and their brains, indicating a potential presence of early-onset cardiac- and neuroinflammation. While preliminary, the results highlight the feasibility for TSPO-PET imaging in the in vivo assessment of chronic inflammation in several organs simultaneously, particularly within a dystrophic disease. A larger longitudinal study across age groups (immature, mature, aged) and DMD disease severity (normal, mild, moderate, severe) using our protocol in same-sex subjects will confirm whether TSPO-PET can track multi-organ activated immune cells to better understand their contribution to dysfunctional outcomes.

## Data Availability

Not applicable.

## Appendix B

**Table A1:** Sample size for the number of unique wild-type and MDX mice successfully completed for each protocol cohort.

	PET	Biodistribution	Autoradiography	Immunohistochemistry	H&E
**Wild-type** ***C57Bl10***	6	2	2	3	1
**MDX** ***mdx:utrn*(+/−)**	4	4	1	3	1
